# Wound specific quality of life after blast or gunshot injury: Validation of the wound QoL instrument

**DOI:** 10.1371/journal.pone.0277094

**Published:** 2022-10-31

**Authors:** Andreas Älgå, Jonas Malmstedt, Ann-Mari Fagerdahl

**Affiliations:** 1 Department of Clinical Science and Education, Södersjukhuset, Karolinska Institutet, Stockholm, Sweden; 2 Department of Global Public Health, Karolinska Institutet, Stockholm, Sweden; Örebro University: Orebro Universitet, SWEDEN

## Abstract

**Background:**

Acute blast or gunshot wounds have a negative effect on the patients’ health related quality of life (HRQoL). No validated instrument exists to assess the HRQoL of patients with such wounds. Therefore, we aimed to test and validate a subscale of an existing HRQoL instrument among patients with acute blast or gunshot wounds.

**Methods:**

We used data from a randomized controlled trial comparing negative pressure wound therapy with standard treatment of civilian adults with acute extremity blast or gunshot wounds. We evaluated the reliability (internal consistency, stability) and validity of the *body* subscale of the Wound QoL instrument using the World Health Organisation 20 question self-reporting questionnaire as gold standard.

**Results:**

A total of 152 participants were included in the study. The participants were predominantly (93.4%) male, and median age was 29.0 years (IQR 21.0–34.0). The internal consistency was acceptable while a test-retest analysis indicated instability in the Wound QoL instrument. The content validity of the instrument was considered satisfactory; however, the criterion validity was found to be insufficient.

**Conclusions:**

Our results indicate that Wound QoL is a promising instrument for the assessment of wound specific HRQoL among patients with acute blast or gunshot wounds. Further testing and validation is needed.

## Introduction

The global number of direct fatalities caused by armed conflict and terrorism is increasing, reaching an estimated 129 700 in 2017 [1]. For each person killed, at least two people are wounded [2]. Injuries caused by blasts or gunshots often result in large wounds, with negative effect on the patient’s health related quality of life (HRQoL) [3, 4]. The extent of this negative effect is unknown. In addition, previous studies on wound specific HRQoL have mainly focused on the long-term effects in patients with hard-to-heal wounds [5, 6], whereas the acute phase has been insufficiently studied.

HRQoL in patients with wounds can be measured either with generic tools or with wound specific tools. One of the first wound specific HRQoL tools was the 47-item Cardiff Wound Impact Schedule (CWIS) [7]. CWIS was designed to measure HRQoL in patients with hard-to-heal wounds and is now widely established. However, patients have deemed the tool too extensive, which has led to participant drop-out in validation studies [8, 9]. Therefore, a new tool, Wound-QoL, was created based on the CWIS and two German instruments [9]. Wound-QoL contains 17 items measuring HRQoL in three domains: body, psyche, and everyday living.

To optimize and individualize the care it is important not only to measure treatment efficacy and safety but to also focus on the patients´ experience of their condition and treatment [10]. There is no validated instrument to assess the HRQoL of patients with acute wounds. Such an instrument could help to increase the understanding of these patients, and aid the development of treatment strategies. Therefore, we aimed to test and validate the Wound-QoL instrument for the assessment of the physical aspects of wound specific HRQoL among patients with acute blast or gunshot wounds.

## Methods

### Study setting and population

We used data from a randomized controlled trial comparing negative pressure wound therapy (NPWT) with standard treatment of adults (≥18 years) with acute (≤72 h) extremity blast or gunshot wounds (ClinicalTrials.gov ID: NCT02444598) [11]. Participants were enrolled at two civilian hospitals in Jordan and Iraq from June 9, 2015, to October 24, 2018. At the end of the first surgical procedure, the participants were randomly assigned to NPWT, involving a commercial NPWT device, or standard treatment, involving wound dressings with non-adhesive sterile gauze, according to the International Committee of the Red Cross war surgery protocol [12]. The groups were well balanced in baseline characteristics, and NPWT did not yield superior clinical outcomes compared with standard treatment [13]. For the present study, we included all randomized patients with blast or gunshot wounds who completed both of the study instruments (described below) at baseline, irrespective of treatment modality. Dedicated research nurses collected and entered the data into paper-based case report forms during the study period.

### Instruments

The Wound QoL instrument is developed and validated to assess disease-specific HRQoL of patients with hard-to-heal wounds [9]. The instrument consists of 17 items on impairment, which are always assessed retrospectively. The items are assigned to the subscales “body”, “psyche”, and “everyday life”. The subscales have been shown to be valid and responsive [14]. In the present study we used items 1–4 in the “body” subscale, 1) my wound hurt, 2) my wound had a bad smell, 3) there was a disturbing discharge from the wound, and 4) the wound has affected my sleep. The “psyche” and “everyday living” subscales were not deemed relevant for acute wounds. Therefore, these subscales were omitted.

The World Health Organisation 20 question self-reporting questionnaire (SRQ-20) was originally developed as a tool to screen for mental disorder [15], and has been used to assess symptom change over time [16]. The instrument is widely used, has a validated Arabic version [17, 18], and is validated for the use in low- and middle-income country emergency settings [19], as well as for longitudinal assessments [20]. In the present study we used the SRQ-20 questions 1, 2, 3, and 7, which measure somatic symptoms [21].

### Reliability

We evaluated the reliability of the “body” subscale of the Wound QoL instrument. To test reliability in regard to internal consistency we estimated the correlation between the four subscale items at baseline by using Cronbach´s alpha coefficient. A Cronbach´s alpha coefficient of 0.7 or higher was considered acceptable [22]. We also tested stability (test-retest reliability) with interclass correlation (ICC) among the participants who had two Wound QoL measurements. An ICC above 70 was considered acceptable [23].

### Validity

We assessed validity according to content validity and criterion validity. Content validity refers to the extent to which a measure covers relevant areas of the condition or disease [24]. Criterion validity compares scores from the instrument in focus to an instrument that targets the same phenomena, and is regarded as gold standard [25]. Content validity was assessed by an expert group following discussions on which of the Wound QoL items could be relevant for the patient group under study. Consensus was reached to exclude the “body” subscale’s item number 5 (the treatment of the wound has been a burden) since this item focuses on the treatment of long-lasting or chronic wounds. The remaining four items were considered relevant for the present patient group with acute wounds. Since this discussion, a revised version of Wound QoL has been published, in which item number 5 has been removed from the “body” subscale [26]. We measured criterion validity by comparing the score of the “body” subscale of the Wound QoL instrument to a total score of the SRQ-20 items 1, 2, 3, and 7, using SRQ-20 as gold standard. The correlation between the two instruments was calculated with Spearman´s rho, with scores between 0.30 and 0.49 indicating acceptable correlation [27].

### Ceiling and floor effect

Ceiling effect occurs when the scores of an instrument reach the maximum possible score. Floor effect occurs when the scores are equal to the minimum possible score. If ceiling or floor effect occurs in 15 percent or more of the questionnaires, it is considered hard to detect improvement or decline in the rated health status [28].

### Statistical analysis

We analysed data using SPSS^®^ Statistics (IBM Corp., Armonk, New York, USA).

### Ethics

This study was part of a larger clinical trial [13], which received ethical approval from the Ethics Review Committee of the Jordan Ministry of Health (MOH REC 150037), the Ethics Review Board of Médecins Sans Frontières (ID 1520), the Research Ethics Committee, Kurdistan Regional Government in Iraq (2:10 6/3/2017), and the Swedish Ethical Review Authority (2019–01975). All participants provided written informed consent.

## Results

A total of 152 participants were included in the study. The participants were predominantly (93.4%) male, and median age was 29.0 years (IQR 21.0–34.0). Some 99/152 (65.1%) participants were smokers, and 13/152 (8.6%) participants had prior diseases, among which one had diabetes mellitus. Wounds in 94/152 (61.8%) participants were caused by gunshots, and in 58/152 (38.2%) participants by bomb blasts. Table 1 summarises the study population characteristics. Some 90/152 (59.2%) participants completed the Wound QoL questionnaire at two occasions, once at baseline and once after 5–7 days.

**Table 1:** Study population.

**Sex**	Male	142 (93.4)
	Female	10 (6.6)
**Age** * **, median (IQR)**		29.0 (21.0–34.0)
**Cause of injury**	Blast	58 (38.2)
	Gunshot	94 (61.8)
**Smoking**	Yes	99 (65.1)
	No	53 (34.9)
**Diabetes mellitus**	Yes	1 (0.7)
	No	151 (99.3)
**Other diseases**	Yes	12 (7.9)
	No	140 (92.1)

IQR, interquartile range.

*One patient missing.

All data are presented as n (%) unless otherwise stated.

### Reliability

The Cronbach´s alpha coefficient was 0.7. Test-retest analysis was performed on questionnaires from 90 participants with an ICC of 0.47.

### Validity

Content validity was discussed within the expert group and the four items constituting the “body” subscale were found to be valid and relevant for the studied patient group. The criterion validity assessments included all 152 participants. Spearman´s rho was 0.20.

### Ceiling and floor effect

We found no signs of ceiling effect as no participant received the highest score. Some 19/152 (12.5%) participants had minimum scores, indicating acceptable levels regarding floor effect.

## Discussion

Our results indicate that the “body” subscale of the Wound QoL instrument could represent a reliable and valid tool for the assessment of physical aspects of wound specific HRQoL among patients with acute blast or gunshot wounds. The internal consistency of the subscale items was acceptable; however, the test-retest analysis indicated instability in the Wound QoL instrument. This instability might be partially explained by a rapid clinical improvement among patients with an acute traumatic wound, compared to a slower change of HRQoL among patients with hard-to-heal wounds. One way to achieve a more reliable test-retest analysis could be to distribute the second questionnaire (re-test) closer in time to the first one.

We demonstrate that the Wound QoL instrument may be able to detect improvement and deterioration in HRQoL since we did not find any ceiling or floor effect. The content validity of the instrument was considered satisfactory by the expert group. However, the criterion validity was found to be insufficient, indicating that the use of SRQ-20 as gold standard for quality of life measures was suboptimal. When designing the study, we opportunistically decided to use SRQ-20 for our analyses as one of the study sites had already implemented the instrument as a standard screening tool. Further, the instrument is well-established and used worldwide in emergency settings in conflict areas. To assess its use in the present analyses was therefore deemed to be of value, in particular considering the lack of instruments aimed at measuring HRQoL in patients with acute wounds.

The impact of acute and traumatic wounds on patients´ HRQoL is scarcely described in the literature. One small study published in 2000 found that the patients with acute wounds rated their overall HRQoL higher compared to the patients with hard-to-heal wounds [29]. The authors concluded that the results might be affected by the temporary condition of the patients with acute wounds as compared to the more chronic condition of the patients with hard-to-heal wounds. It should be noted that the instrument used in the study had not been validated for patients with acute wounds.

### Limitations

Some limitations should be considered. First, the sample size is rather small. However, to prospectively include study participants with acute blast or gunshot wounds is a challenge. Particularly at low-resource or conflict settings. Second, only the “body” subscale of the Wound QoL instrument was used. However, this was the only of the three subscales that was considered relevant. The main limitation is the use of SRQ-20 as gold standard. The SRQ-20 instrument is mainly used as a screening tool for mental disorder. Still, we anticipated that the instrument items measuring somatic symptoms would prove to be transferrable to the physical aspects of HRQoL. The low criterion validity indicates that this might not be the case. Bearing the above mentioned limitations in mind, we still believe that the present study adds important knowledge on the utility and limitations of the SRQ-20 instrument, considering its widespread use.

## Conclusions

The “body” subscale of the Wound QoL is a promising instrument for the assessment of wound specific HRQoL among patients with acute blast or gunshot wounds. Future research in patients with acute traumatic wounds could be aimed at how to differentiate between the HRQoL effect generated by the psychological trauma and the effect generated by the actual wound.
